# Allelic Variants of the *DPYD* Gene in Russian Patients with Cancer: The Results of Exome Sequencing

**DOI:** 10.3390/genes17091082

**Published:** 2026-09-09

**Authors:** Denis Fedorinov, Vladimir Lyadov, Marina Lyadova, Sherzod Abdullaev, Ivan Sychev, Anna Filatova, Lavrentii Danilov, Oleg Glotov, Iuliia Budagova, Karin Mirzaev, Dmitry Sychev

**Affiliations:** 1 Federal State Budgetary Research Institution «Russian Research Center of Surgery Named After Academician B.V. Petrovsky», 119991 Moscow, Russia; sherzodx5@gmail.com (S.A.); sychev_iv@bk.ru (I.S.); karin05doc@yandex.ru (K.M.); dimasychev@mail.ru (D.S.); 2 Moscow Multidisciplinary Clinical Center “Kommunarka”, 108814 Moscow, Russia; 3 Federal State Budgetary Educational Institution of Further Professional Education “Russian Medical Academy of Continuous Professional Education”, Ministry of Healthcare of the Russian Federation, 125993 Moscow, Russia; vlyadov@gmail.com; 4 Moscow State Budgetary Healthcare Institution “Oncological Center No. 1 of Moscow City Hospital Named After S.S. Yudin, Moscow Healthcare Department”, 115446 Moscow, Russia; dr.lyadova@gmail.com; 5 Novokuznetsk State Institute of Postgraduate Medical Education—Branch of Russian Medical Academy of Continuous Professional Education, 654005 Novokuznetsk, Russia; 6 E.M. Tareev Clinic of Rheumatology, Nephrology, and Occupational Pathology, I.M. Sechenov First Moscow State Medical University (Sechenov University), 119991 Moscow, Russia; filatovaanet@mail.ru; 7 Faculty of Fundamental Medicine, Lomonosov Moscow State University, 119991 Moscow, Russia; 8 Department of Genetics and Biotechnology, Federal State Budgetary Educational Institution of Higher Education, St. Petersburg State University, 7-9 Universitetskaya Nab., 199034 Saint Petersburg, Russia; l.danilov@spbu.ru; 9 Federal Scientific and Clinical Center for Infectious Diseases, Federal Medical Biological Agency of Russian Federation, 9A Professora Popova Str., 197022 Saint Petersburg, Russia; olglotov@mail.ru (O.G.); yulia.zaitzeva674@yandex.ru (I.B.); 10 State Budgetary Healthcare Institution “Moscow Scientific and Practical Center for Laboratory Research of the Moscow Healthcare Department”, 49 Orekhovy Boulevard, Bld. 1, 115580 Moscow, Russia

**Keywords:** gene, genome, genetics, genotype, pharmacogenetics, pharmacogenomics, *DPYD*, cancer, whole-exome sequencing

## Abstract

**Background/Objectives**: Pharmacogenetic testing of the dihydropyrimidine dehydrogenase gene (*DPYD*) is increasingly incorporated into clinical practice to identify patients at increased risk of fluoropyrimidine-related toxicity. However, most routine assays target a limited number of well-established variants, whereas the *DPYD* gene demonstrates substantial population variability and may harbor rare potentially functional alleles that are not detected by conventional targeted testing. Data describing coding and splice-region *DPYD* variants detectable by whole-exome sequencing in Russian oncology populations remain limited. This study aimed to characterize the frequency and distribution of common, clinically relevant, and rare *DPYD* variants in a Russian cohort of patients receiving fluoropyrimidine-containing chemotherapy and to compare the observed allele frequencies with European and East Asian reference populations. **Methods**: Descriptive pharmacogenetic analysis was performed in 339 patients with malignant tumors treated with fluorouracil, leucovorin, oxaliplatin, and docetaxel (FLOT), folinic acid, fluorouracil, and oxaliplatin (FOLFOX), or folinic acid, fluorouracil, irinotecan, and oxaliplatin (FOLFIRINOX) regimens. Whole-exome sequencing was performed using Illumina technology with exome enrichment by KAPA HyperExome and a sequencing depth of at least 100×. Sequence reads were aligned to the Genome Reference Consortium Human Build 38 (GRCh38) reference genome, germline variants were called using Genome Analysis Toolkit (GATK), HaplotypeCaller, and functional annotation was performed with Ensembl Variant Effect Predictor. *DPYD* variants were classified according to their population frequency, predicted functional effect, ClinVar annotations, and current pharmacogenetic recommendations. Allele and genotype frequencies were calculated and descriptively compared with Genome Aggregation Database (gnomAD) v4.1.1 Non-Finnish European and East Asian populations. **Results**: Seventeen *DPYD* variants were identified. The most frequent alternative alleles were rs1801265 (24.93%), rs1801159 (17.70%), rs2297595 (11.06%), rs1801160 (7.08%), and rs17376848 (5.16%). Their distribution was generally closer to that observed in the Non-Finnish European population than in East Asian populations. The established reduced-function variant rs67376798 (c.2846A>T, p.Asp949Val) was detected in one heterozygous patient, corresponding to a carrier frequency of 0.29% and an allele frequency of 0.15%. The HapB3 proxy variant rs56038477 (c.1236G>A) was identified in 13 heterozygous patients, with a carrier frequency of 3.83% and an allele frequency of 1.92%; confirmation of the functional intronic variant rs75017182 would be required for definitive HapB3 assignment. Overall, rs67376798 or rs56038477 was detected in 14 patients (4.13%). In addition, rare variants with a cohort allele frequency below 1% were identified in 11 patients (3.24%). Among these, p.Thr65Ala, p.Thr65Met, p.Asn151Asp, and p.Val691Leu represented potentially relevant findings requiring further functional validation. **Conclusions**: Whole-exome analysis revealed a heterogeneous spectrum of *DPYD* variants in the studied Russian oncology cohort, including both established pharmacogenetic markers and rare variants that would not be captured by limited targeted panels. The overall allele-frequency pattern was predominantly similar to that of European reference populations, although several rare variants demonstrated distinct distributions. These findings support the value of population-specific characterization of *DPYD* and suggest that expanded sequencing approaches may complement conventional pharmacogenetic testing by identifying rare potentially functional alleles.

## 1. Introduction

The dihydropyrimidine dehydrogenase gene (*DPYD*), located on chromosome 1p21.3, encodes dihydropyrimidine dehydrogenase (DPD), a key enzyme in the pyrimidine catabolic pathway. DPD catalyzes the first and rate-limiting step in the degradation of the endogenous pyrimidines uracil and thymine and also plays a central role in the catabolism of fluoropyrimidines. Approximately 80% of administered 5-fluorouracil is degraded through the DPD-dependent pathway; consequently, reduced DPD activity may result in increased systemic fluoropyrimidine exposure and a substantially increased risk of severe or potentially life-threatening toxicity. *DPYD* is highly polymorphic, and its genetic variability includes common polymorphisms, as well as rare coding, splice-region, and intronic variants. Although a limited number of *DPYD* variants have well-established effects on enzyme activity and fluoropyrimidine toxicity, the functional and clinical significance of many rare variants remains insufficiently characterized. This extensive allelic heterogeneity provides an important rationale for investigating a broader spectrum of *DPYD* variants beyond those included in conventional targeted pharmacogenetic panels [1].

In recent years, pre-treatment testing for dihydropyrimidine dehydrogenase (DPD) deficiency has increasingly been incorporated into routine clinical practice in many countries. In 2020, the European Medicines Agency recommended genotypic and/or phenotypic assessment of DPD status before initiating systemic therapy with 5-fluorouracil, capecitabine, or tegafur [2]. In the United Kingdom, the Medicines and Healthcare products Regulatory Agency recommended that all patients undergo testing before starting systemic fluoropyrimidine therapy, and screening for common *DPYD* variants was incorporated into the national genomic medicine service [3]. Recommendations for genotype-guided fluoropyrimidine dosing have also been developed by the Dutch Pharmacogenetics Working Group [4], as well as by professional societies in Spain [5]. These developments reflect the transition of *DPYD* pharmacogenetic testing from a predominantly research-based approach to routine clinical practice in oncology [6,7].

However, the clinical interpretation of test results should not be limited to determining the presence or absence of an individual allelic variant. According to the Clinical Pharmacogenetics Implementation Consortium (CPIC) guidelines, each *DPYD* allele is assigned an activity value: 1 for normal function, 0.5 for decreased function, and 0 for no function. The activity score is calculated from the combined values of the two alleles and is used to infer whether patient’s metabolizer phenotype is normal, intermediate, or poor [8]. Translating genotyping results into predicted enzyme activity is essential for generating a clinically meaningful interpretation and selecting an appropriate starting dose of a fluoropyrimidine. In selected cases, genotyping may be complemented by phenotyping based on the measurement of endogenous uracil concentrations or the dihydrouracil-to-uracil ratio. However, genotypic and biochemical approaches capture different aspects of interindividual variability in DPD activity [9].

In 2024, the Association for Molecular Pathology, in collaboration with the American College of Medical Genetics and Genomics, CPIC, the College of American Pathologists, the Dutch Pharmacogenetics Working Group, and other professional organizations, proposed a standardized panel of *DPYD* variants for use in clinical laboratories [10]. The minimum Tier 1 panel included c.1905+1G>A, c.1679T>G, c.1129-5923C>G, c.557A>G, c.868A>G, c.2279C>T, and c.2846A>T. Selection of these variants was based on their functional relevance, population frequency, availability of clinical evidence and reference materials, and technical feasibility of reliable detection. The most extensively studied variants remain c.1905+1G>A (DPYD*2A, rs3918290), c.1679T>G (DPYD*13, rs55886062), c.2846A>T (rs67376798), and the deep intronic variant c.1129-5923C>G (rs75017182), which is the functional component of the HapB3 haplotype. Tier 2 comprises an expanded panel of potentially relevant alleles. Classification within this tier indicates possible functional or clinical relevance but does not imply that each variant is supported by the same level of evidence as the most extensively characterized Tier 1 variants. Many Tier 2 variants are exceedingly rare, have been identified in only a small number of carriers, or are supported by limited and, in some cases, conflicting functional evidence.

Most polymerase chain reaction (PCR)-based panels currently used in clinical practice target a limited number of preselected variants. This approach offers rapid turnaround, relatively low cost, and straightforward interpretation but cannot detect rare or previously unreported variants located elsewhere in the gene. Consequently, increasing attention is being given to extended sequencing of the *DPYD* coding regions and adjacent splice-site regions. Recent studies using extended *DPYD* sequencing have demonstrated that rare coding and splice-region variants may contribute to interindividual variability in DPD activity and fluoropyrimidine toxicity and may therefore complement conventional targeted genotyping [11,12,13].

The aim of this study was to characterize the spectrum of *DPYD* allelic variants, determine genotype and alternative allele frequencies, assess the prevalence of clinically relevant, potentially functional, and rare variants in a Russian cohort of patients with cancer treated with the fluoropyrimidine-containing FLOT, FOLFOX, or FOLFIRINOX regimens, and compare the observed frequencies with reference data from European and Asian populations. To the best of our knowledge, this is the first study conducted in the Russian Federation to use whole-exome sequencing to comprehensively characterize common, clinically relevant, and rare *DPYD* variants in patients with cancer receiving these fluoropyrimidine-containing regimens.

## 2. Materials and Methods

A prospective observational study was conducted at the Department of Chemotherapy No. 1, S.S. Yudin City Clinical Hospital, Moscow, between 2020 and 2023. Patients receiving systemic anticancer therapy were consecutively enrolled in the study. At the time of inclusion, peripheral blood samples were collected from each patient and stored under frozen conditions until further genetic analysis.

After completion of patient enrollment and clinical data collection, the stored blood samples were transferred to the sequencing laboratory. The sequencing cohort included 339 patients treated with the FOLFOX, FOLFIRINOX, or FLOT chemotherapy regimen. The mean age was 64.5 ± 10.3 years, with a median of 66 years (interquartile range, 59–72 years); patient age ranged from 32 to 84 years. Men comprised the majority of the cohort (192 patients, 56.6%). Most patients received FOLFOX (257, 75.8%), whereas 45 (13.3%) received FOLFIRINOX and 37 (10.9%) received FLOT. Detailed baseline clinical characteristics of the study population are presented in Supplementary Table S1.

### 2.1. Whole-Exome Sequencing

Genetic analysis was performed using whole-exome sequencing (WES). Exome libraries were prepared, and protein-coding regions were enriched using the KAPA HyperExome system. Sequencing was performed on an Illumina platform to generate 150-bp paired-end reads at a mean coverage depth of 100×. Initial quality control of the raw sequencing files in FASTQ format was performed using FastQC (v0.12.1) [14]. Adapter sequences and low-quality regions were removed using fastp (v1.3.6) [15]. Following preprocessing, reads were aligned to the GRCh38.p14 human reference genome using Burrows–Wheeler Aligner with Maximal Exact Matches (BWA-MEM) (v0.7.19). The resulting alignment files were sorted and indexed using Sequence Alignment/Map tools (SAMtools) (v1.23) [16]. Duplicate reads were marked using the MarkDuplicates tool from Picard (v3.4.0) (Picard2019toolkit, Broad Institute, 2019. https://broadinstitute.github.io/picard/ accessed on 7 May 2026). Base quality scores were subsequently recalibrated using the BaseRecalibrator and ApplyBQSR tools from GATK (v4.6.2.0) [17].

### 2.2. Genetic Variant Calling and Filtering

Variant calling, joint genotyping, and hard filtering were performed based on the GATK Best Practices recommendations [18,19].

Germline single-nucleotide variants (SNVs) and short insertions and deletions (indels) were called separately for each sample using the HaplotypeCaller tool from GATK (v4.6.2.0) in Variant Call Format (VCF), specifically genomic VCF (gVCF), mode. Variant calling was restricted to the target regions of the exome capture panel. Individual gVCF files were imported into a GenomicsDB datastore using GenomicsDBImport (GATK v4.6.1.0), followed by joint genotyping with GenotypeGVCFs (GATK v4.6.1.0).

SNVs and indels were separated using GATK SelectVariants and hard-filtered using VariantFiltration. Variants were flagged as low-quality if they met any of the following criteria: Quality by Depth (QD) < 2.0, Fisher Strand bias (FS) > 60.0, or Mapping Quality (MQ) < 40.0 for SNVs; and QD < 2.0, FS > 200.0 for indels.

The filtered SNV and indel datasets were merged and normalized against the GRCh38.p14 reference sequence using BCFtools (v1.23) [16]. Multiallelic records were split into separate biallelic records, and only variants assigned a PASS filter status were retained for subsequent analysis. At the genotype level, calls with a sequencing depth below 20 reads (DP < 20) or a genotype quality below 20 (genotype quality (GQ) < 20) were set to missing. Variants with more than 30% missing genotype calls were excluded.

Hardy–Weinberg equilibrium (HWE) was assessed for each identified *DPYD* variant using a two-sided exact test implemented in the HWExact function of the HardyWeinberg package in R. A *p*-value below 0.05 was considered indicative of a statistically significant deviation from Hardy–Weinberg equilibrium.

### 2.3. Extraction and Annotation of DPYD Variants

Variants located within the *DPYD* gene were extracted from the complete exome dataset for the present study. Functional and population-level annotation was performed using Ensembl Variant Effect Predictor (VEP) (v116.0) [20] against the GRCh38.p14 human reference genome assembly. Annotation sources included RefSeq, Matched Annotation from NCBI and EMBL-EBI (MANE) Select, dbNSFP version 5.3a [21], ClinVar (Release: 2 March 2026), gnomAD, the 1000 Genomes Project, UniProt, and Consensus Coding Sequence (CCDS).

*DPYD* variants were described according to the reference transcript NM_000110.4 and protein sequence NP_000101.2. For each variant, the genomic position, reference and alternative alleles, reference single-nucleotide polymorphism identifier (rsID), predicted molecular consequence, Human Genome Variation Society nomenclature for coding DNA (HGVSc) and protein sequences (HGVSp), patient genotype, sequencing depth, and numbers of reads supporting the reference and alternative alleles were recorded.

The potential functional relevance of the identified variants was assessed using ClinVar annotations and predictions generated by computational algorithms, including Combined Annotation Dependent Depletion (CADD) [22], Rare Exome Variant Ensemble Learner (REVEL) [23], Sorting Intolerant From Tolerant (SIFT), Polymorphism Phenotyping version 2 (PolyPhen-2), and MutationTaster. Evolutionary conservation metrics, including phastCons, phyloP, and Genomic Evolutionary Rate Profiling (GERP), were also considered. Population frequencies were obtained from gnomAD [24], including ancestry-specific data for the non-Finnish European population.

Clinical interpretation of *DPYD* variants was based on current recommendations from the Clinical Pharmacogenetics Implementation Consortium (CPIC) and the joint recommendations issued by the Association for Molecular Pathology and collaborating professional organizations regarding the composition and interpretation of *DPYD* pharmacogenetic panels. Variants were classified as clinically relevant variants associated with established decreased function, proxy variants for clinically relevant haplotypes, variants of potential research interest, or variants of uncertain or undetermined clinical significance. Variants with an alternative allele frequency below 1% in the study cohort were classified as rare.

## 3. Results

### 3.1. Overall Summary of Genotyping Results

Whole-exome analysis identified 17 allelic variants in *DPYD*, including common polymorphisms, rare missense variants, synonymous substitutions, and one variant of established clinical relevance.

For 15 of the 17 variants, genotypes were successfully determined in all 339 patients. Genotyping of rs761479700 was informative in 335 patients, corresponding to a call rate of 98.82%. For rs45589337, genotype data were available for 308 patients, yielding a call rate of 90.86%. The frequency of this variant was calculated using only patients with successfully determined genotypes.

Analysis of genotype distributions showed that all investigated *DPYD* variants were consistent with Hardy–Weinberg equilibrium, with no statistically significant deviations observed within the study cohort (*p* > 0.05).

At least one alternative *DPYD* allele was identified in 258 of the 339 patients, accounting for 76.11% of the study cohort. However, this proportion was largely driven by the high prevalence of common variants with normal or undetermined functional activity and should not be interpreted as the prevalence of genetically determined DPD deficiency.

### 3.2. DPYD Genotype and Allele Frequencies

The most common variant was rs1801265, c.85T>C, p.Cys29Arg. The alternative allele was detected in 145 patients, including 121 heterozygous and 24 homozygous carriers, with an allele frequency of 24.93%.

The second most common variant was rs1801159, c.1627A>G, p.Ile543Val. The alternative allele was identified in 105 patients, of whom 90 were heterozygous and 15 were homozygous. Its frequency was 17.70%. The rs2297595 variant, c.496A>G, p.Met166Val, was detected in 74 patients. Of these, 73 were heterozygous, whereas one was homozygous for the alternative allele. The alternative allele frequency was 11.06%. The rs1801160 variant, c.2194G>A, p.Val732Ile, was identified in 45 patients, including three alternative-allele homozygotes, with an alternative allele frequency of 7.08%.

The synonymous variant rs17376848, c.1896T>C, p.Phe632=, was identified in 35 patients, all of whom were heterozygous. Its alternative allele frequency was 5.16%. All remaining variants had allele frequencies below 2%. Genotype distributions and mutant allele frequencies are presented in Table 1.

## 4. Discussion

### 4.1. Comparison with European and Asian Population Data

For descriptive comparisons and potential population differences, allele frequencies from the non-Finnish European and East Asian populations in gnomAD v4.1.1 (https://gnomad.broadinstitute.org) were used.

The frequencies of the most common variants in the study cohort were closer to those reported for the non-Finnish European population. For example, the alternative allele frequency of rs1801265 was 24.93%, compared with 21.9% in the European population and 5.39% in the East Asian population. For rs2297595, the corresponding frequencies were 11.06% in the study cohort, 9.38% in the European population, and 1.77% in the East Asian population. The frequency of rs1801160 reached 7.08% in the study cohort, exceeding the frequencies reported for the European and East Asian populations, which were 4.5% and 1.86%, respectively.

A different pattern was observed for rs1801159. Its alternative allele frequency in the study cohort was 17.70%, which was close to the European frequency of 19.95% but lower than the 26.94% reported for the East Asian population. The synonymous variant rs17376848 had a frequency of 5.16% in the study cohort, comparable to the European estimate of approximately 4.52%; however, its frequency may reach 12.98% in some East Asian datasets.

The HapB3 proxy variant rs56038477 had an alternative allele frequency of 1.92%, similar to the 2.20% reported for the non-Finnish European population. In the East Asian population, this variant is rarely detected, with a frequency below 1%.

**Figure 1:**
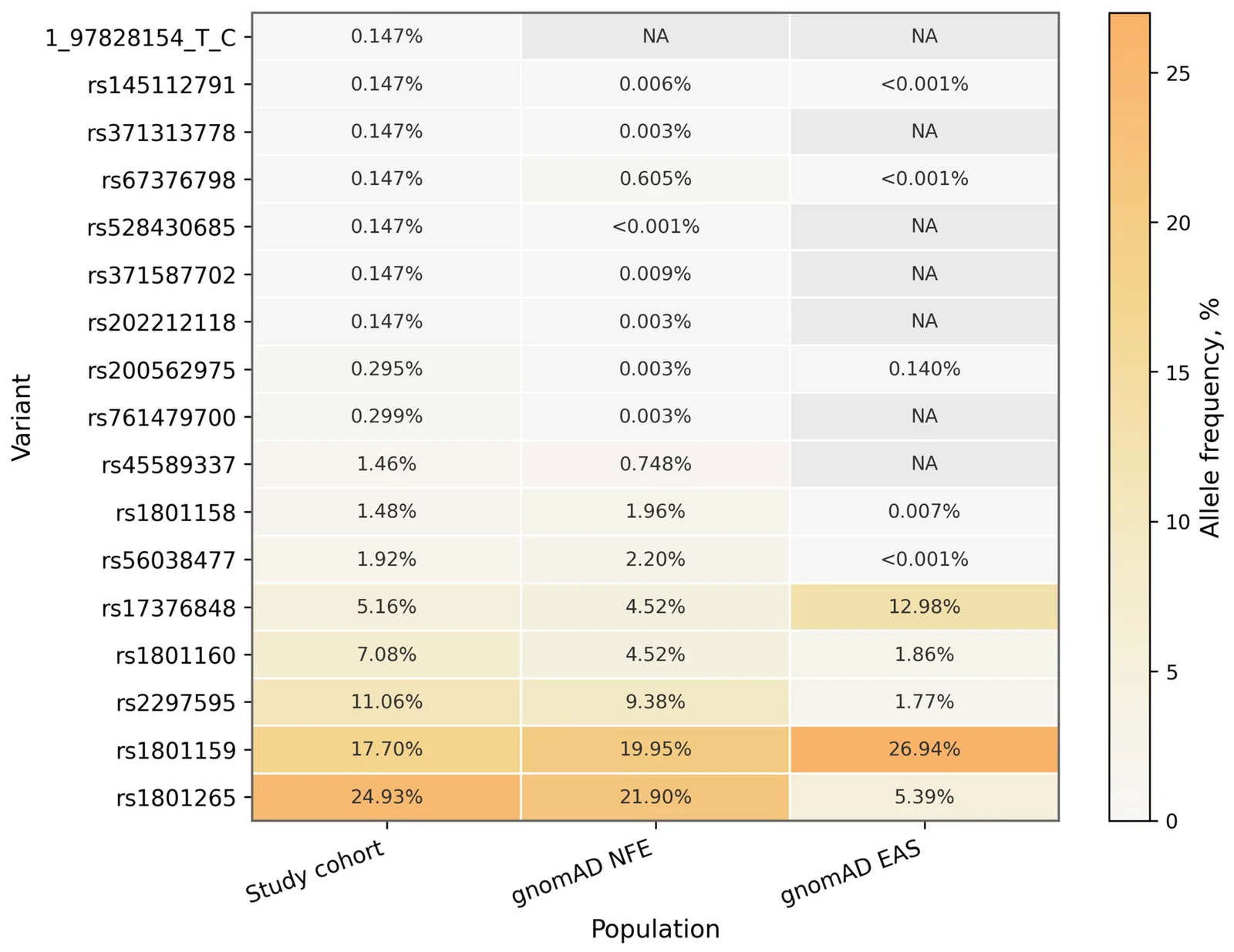
Comparison of *DPYD* allelic variant frequencies between the study cohort and the non-Finnish European and East Asian populations. The heatmap shows the alternative allele frequencies of the 17 *DPYD* variants identified in the study cohort and in the gnomAD v4.1.1 reference populations: non-Finnish European (NFE) and East Asian (EAS). Values are expressed as percentages. Variants are ordered by increasing alternative allele frequency in the study cohort. Color intensity corresponds to allele frequency, with lighter shades indicating lower frequencies and darker shades indicating higher frequencies. Variants for which an exact frequency was unavailable in the corresponding reference population are shown in gray and labeled NA.

Overall, the distribution of common and clinically relevant *DPYD* variants in the study cohort largely resembled the European population profile. The most pronounced differences between European and East Asian populations were observed for rs1801265, rs2297595, rs1801160, rs56038477, and rs67376798, which were substantially more common among individuals of European ancestry. In contrast, rs1801159 was more prevalent in the East Asian population. The observed differences are consistent with the marked ancestry-dependent variability of the *DPYD* variant spectrum described in previous population studies and systematic analyses, emphasizing that panels developed predominantly in European populations may not capture the full spectrum of potentially relevant variants in other ancestry groups [25]. Differences in the frequencies of individual rare variants should be interpreted cautiously because of the small number of carriers and the absence of a population-based control group for direct comparison. Figure 1 compares the frequencies of *DPYD* allelic variants identified in the study cohort with reference data from the non-Finnish European and East Asian populations.

### 4.2. Clinically Relevant Variants

The rs67376798 variant, c.2846A>T, p.Asp949Val, was identified in the heterozygous state in one patient. The carrier prevalence was 0.295%, and the alternative allele frequency was 0.147%. rs67376798 is an established decreased-function variant associated with reduced DPD activity [26]. It is considered when assigning the DPD metabolizer phenotype and selecting the initial fluoropyrimidine dose according to CPIC guidelines and recommendations from other professional organizations [8].

The synonymous variant rs56038477, c.1236G>A, p.Glu412=, was identified in the heterozygous state in 13 patients. The carrier prevalence was 3.83%, and the alternative allele frequency was 1.92%.

The rs56038477 variant has traditionally been used as a proxy marker for the HapB3 haplotype. However, the deep intronic variant rs75017182, c.1129-5923C>G, which disrupts *DPYD* splicing, is considered the functionally relevant component of this haplotype. Although rs56038477 and rs75017182 are in strong linkage disequilibrium, their linkage is not complete. Current recommendations for standardized genotyping therefore include direct testing for rs75017182, as the isolated detection of rs56038477 does not invariably confirm the presence of the functional HapB3 haplotype [10].

Thus, these 13 patients should be classified as carriers of the HapB3 proxy variant rather than as carriers of a confirmed functional decreased-activity variant. Definitive pharmacogenetic classification would require direct testing for rs75017182 or haplotype analysis.

Overall, either rs67376798 or rs56038477 was identified in 14 patients. The combined carrier prevalence of an established decreased-function variant or the HapB3 proxy variant was 4.13%. No patient carried both rs67376798 and rs56038477.

Other major clinically relevant variants, including rs3918290, c.1905+1G>A, and rs55886062, c.1679T>G, were not detected in the study cohort.

### 4.3. Rare and Potentially Relevant Variants

Using an alternative allele frequency threshold of <1%, rare variants were identified in 11 of the 339 patients (3.24%). Most rare variants were detected in the heterozygous state. The findings are presented in Table 2.

The highest CADD score, 32.0, was observed for rs371587702, c.194C>T, p.Thr65Met. Another variant affecting the same amino acid residue, 1_97828154_T_C, c.193A>G, p.Thr65Ala, had a CADD score of 26.9. Both variants were identified in the heterozygous state in different patients. The detection of two distinct substitutions at the Thr65 residue is of particular research interest; however, without functional assessment of enzyme activity, this finding alone does not establish the clinical relevance of this region.

The c.193A>G, p.Thr65Ala variant warrants particular attention because, at the time of analysis, no corresponding rsID or records in major clinical and pharmacogenetic databases could be identified. It may therefore represent a previously unreported rare *DPYD* variant. A high CADD PHRED score suggesting potential deleteriousness, together with its location at the same amino acid residue as the known rare p.Thr65Met variant, makes p.Thr65Ala a promising candidate for further functional investigation. Nevertheless, the absence of data on its frequency in independent populations and of experimental evidence regarding its effect on DPD activity precludes any conclusions regarding pathogenicity or its use in clinical decision-making at this stage.

The rs200562975 variant, c.451A>G, p.Asn151Asp, was identified in two patients. Its frequency in the study cohort was higher than that reported for the European reference population but comparable to the frequency observed in the Asian population. Available evidence regarding its functional effect is conflicting, and its clinical significance remains uncertain.

The rs202212118 (p.Val691Leu), rs371313778 (p.Val812Ile), and rs145112791 (p.Leu312Phe) variants were each identified in a single patient. Their low population frequencies and relatively high CADD scores support their consideration as candidates for further functional investigation, but not as established pharmacogenetic markers.

It should be emphasized that CADD, SIFT, PolyPhen, and other computational tools predict the potential effect of a substitution on protein structure or function but do not establish reduced DPD activity. Rare variants with uncertain or conflicting interpretations should not be used automatically to guide reductions in the initial fluoropyrimidine dose. Instead, they should be regarded as candidates for further investigation. Reproducible functional or clinical evidence is required before such findings can inform clinical management.

### 4.4. Variants of Potential Research Interest Without Established Clinical Utility

The rs1801160 (c.2194G>A, p.Val732Ile), rs2297595 (c.496A>G, p.Met166Val), and rs1801158 (c.1601G>A, p.Ser534Asn) variants have previously been investigated as potential modifiers of DPD activity or the risk of adverse reactions. Although some studies have reported associations with altered enzyme activity or fluoropyrimidine tolerability, these findings have not been consistently replicated.

For rs2297595, certain haplotype configurations have been associated with a lower dihydrouracil-to-uracil ratio, a marker of DPD activity. However, the magnitude of this functional effect was considerably smaller than that observed for the established variants rs3918290, rs55886062, rs67376798, and rs75017182 [27].

According to current CPIC guidelines, the presence of rs1801160, rs2297595, or rs1801158 alone does not justify modification of the initial fluoropyrimidine dose. Thus, the relatively high frequencies of rs2297595 and rs1801160 in the Russian cohort are of population-level and research interest; however, these variants cannot currently be classified as established clinically actionable *DPYD* alleles.

### 4.5. Variants Without Established Clinical Significance

The rs1801265 (c.85T>C, p.Cys29Arg) and rs1801159 (c.1627A>G, p.Ile543Val) variants accounted for the largest proportion of alternative allele carriers in the cohort. A substantial number of patients carried at least one of these variants. Nevertheless, large cohort studies have not demonstrated a reproducible association between either variant and a clinically meaningful reduction in DPD activity or an increased risk of fluoropyrimidine-related toxicity [28,29,30,31].

In the CPIC guidelines, rs1801265 and rs1801159 are classified as normal-function variants. Their presence does not affect the calculation of the DPD activity score and does not warrant a reduction in the initial fluoropyrimidine dose.

The synonymous variants rs17376848 (c.1896T>C, p.Phe632=) and rs528430685 (c.1228C>A, p.Arg410=) do not alter the amino acid sequence of the enzyme. Available annotations provided no convincing evidence that either variant independently affects DPD activity. Therefore, they were not classified as clinically relevant pharmacogenetic markers.

The rs56038477 variant should be considered separately. Although it is also synonymous, its relevance arises not from a direct alteration of the protein but from its use as a proxy marker for the HapB3 haplotype. It should therefore not be grouped with other neutral synonymous variants; however, its clinical interpretation requires confirmation by direct testing for rs75017182.

### 4.6. Limitations

This study has several limitations. First, its design was primarily descriptive and was aimed at characterizing the spectrum and frequency of *DPYD* variants rather than establishing genotype–phenotype associations.

Second, the cohort included patients with different gastrointestinal malignancies treated with several fluoropyrimidine-containing regimens. The sizes of individual tumor-specific subgroups were unequal, and many rare *DPYD* variants were detected in only one or a few patients. Consequently, the study was not sufficiently powered to evaluate associations between specific *DPYD* variants and particular cancer types, and any apparent differences between tumor subgroups should be considered descriptive only.

Third, the study population was derived from an oncology cohort rather than from a population-based sample. Therefore, the observed allele frequencies should not be interpreted as representative of the entire Russian population. In addition, Russia is characterized by substantial population heterogeneity, while detailed genetic ancestry data were not available for individual participants. Comparisons with gnomAD non-Finnish European and East Asian reference populations should therefore be regarded as descriptive rather than as direct ancestry-matched comparisons.

Fourth, although whole-exome sequencing enables the detection of a broad range of coding and splice-region variants, it has inherent limitations for the assessment of deep intronic and other noncoding variants. In particular, the functionally relevant HapB3 variant rs75017182 (c.1129-5923C>G) is located in a deep intronic region and was not directly assessed by the exome-based approach used in this study. Accordingly, detection of the synonymous proxy variant rs56038477 cannot by itself establish the presence of the functional HapB3 haplotype.

Finally, the functional significance of several rare variants identified in this study remains uncertain. Their potential relevance was assessed mainly using population frequency data, database annotations, and in silico prediction tools such as CADD, REVEL, SIFT, and PolyPhen-2. These approaches can prioritize variants for further investigation but cannot establish reduced DPD activity or clinical actionability. Functional assays, replication in independent cohorts, and prospective genotype–phenotype studies will therefore be required before these rare variants can be incorporated into clinical decision-making.

## 5. Conclusions

In the Russian oncology cohort examined in this study, the *DPYD* variant spectrum was dominated by the common alleles rs1801265, rs1801159, rs2297595, rs1801160, and rs17376848. Overall, their frequencies were closer to those reported for the non-Finnish European population than to those observed in East Asian reference datasets, further demonstrating the marked population variability of *DPYD*.

Clinically relevant decreased-function variants were uncommon. rs67376798 was identified in one patient, whereas the HapB3 proxy variant rs56038477 was detected in 13 patients. Their combined carrier prevalence was 4.13%. However, rs56038477 should be interpreted with caution and, whenever possible, confirmed by direct testing for the functional variant rs75017182.

Rare allelic variants identified in 11 patients were of particular interest. Among these, p.Thr65Ala, p.Thr65Met, p.Asn151Asp, and p.Val691Leu appear to be the most promising candidates for further investigation. At present, their clinical significance remains uncertain, and these variants should not be used independently to guide fluoropyrimidine dose modification.

These findings demonstrate that extended *DPYD* analysis can characterize both common population polymorphisms and rare, potentially functional variants that may be missed by limited PCR-based panels. This highlights the need for further population-specific investigation of the complete *DPYD* variant spectrum, accompanied by the accumulation of functional and clinical evidence for rare alleles.

## Figures and Tables

**Table 1:** *DPYD* Genotyping Results and Allelic Variant Frequencies.

Variant (rsID)	Nucleotide Substitution	Protein Change	Number of Genotyped Patients	Ref/Ref, *n* (%)	Ref/Alt, *n* (%)	Alt/Alt, *n* (%)	Alternative Allele Frequency, %	HWE Exact *p*-Value
No rsID assigned	c.193A>G	p.Thr65Ala	339	338 (99.71)	1 (0.29)	0	0.147	1.000
rs145112791	c.934C>T	p.Leu312Phe	339	338 (99.71)	1 (0.29)	0	0.147	1.000
rs17376848	c.1896T>C	p.Phe632=	339	304 (89.68)	35 (10.32)	0	5.162	1.000
rs1801158	c.1601G>A	p.Ser534Asn	339	329 (97.05)	10 (2.95)	0	1.475	1.000
rs1801159	c.1627A>G	p.Ile543Val	339	234 (69.03)	90 (26.55)	15 (4.42)	17.699	0.133
rs1801160	c.2194G>A	p.Val732Ile	339	294 (86.73)	42 (12.39)	3 (0.88)	7.080	0.226
rs1801265	c.85T>C	p.Cys29Arg	339	194 (57.23)	121 (35.69)	24 (7.08)	24.926	0.386
rs200562975	c.451A>G	p.Asn151Asp	339	337 (99.41)	2 (0.59)	0	0.295	1.000
rs202212118	c.2071G>T	p.Val691Leu	339	338 (99.71)	1 (0.29)	0	0.147	1.000
rs2297595	c.496A>G	p.Met166Val	339	265 (78.17)	73 (21.53)	1 (0.29)	11.062	0.099
rs371313778	c.2434G>A	p.Val812Ile	339	338 (99.71)	1 (0.29)	0	0.147	1.000
rs371587702	c.194C>T	p.Thr65Met	339	338 (99.71)	1 (0.29)	0	0.147	1.000
rs45589337	c.775A>G	p.Lys259Glu	308	299 (97.08)	9 (2.92)	0	1.461	1.000
rs528430685	c.1228C>A	p.Arg410=	339	338 (99.71)	1 (0.29)	0	0.147	1.000
rs56038477	c.1236G>A	p.Glu412=	339	326 (96.17)	13 (3.83)	0	1.917	1.000
rs67376798	c.2846A>T	p.Asp949Val	339	338 (99.71)	1 (0.29)	0	0.147	1.000
rs761479700	c.802C>G	p.Leu268Val	335	333 (99.40)	2 (0.60)	0	0.299	1.000

Note: Genotype percentages were calculated relative to the number of patients with a successfully determined genotype for the corresponding variant. The alternative allele frequency was calculated as the number of alternative alleles divided by twice the number of successfully genotyped patients. Abbreviations: Ref, reference allele; Alt, alternative allele; HWE, Hardy–Weinberg equilibrium; rsID, reference single-nucleotide polymorphism identifier.

**Table 2:** Rare and Potentially Functionally Relevant *DPYD* Variants.

Allelic Variant	Protein Change	Number of Carriers	Mutant Allele Frequency, %	ClinVar Annotation in the Source Data	CADD	Interpretation
No rsID assigned	p.Thr65Ala	1	0.147	Not available	26.9	Rare variant with a potential functional effect
rs145112791	p.Leu312Phe	1	0.147	Not available	24.7	Rare variant with limited clinical evidence
rs200562975	p.Asn151Asp	2	0.295	Conflicting classifications	27.2	Variant of uncertain clinical significance
rs202212118	p.Val691Leu	1	0.147	Uncertain significance	26.6	Variant of uncertain clinical significance.
rs371313778	p.Val812Ile	1	0.147	Not available	23.3	Rare variant with limited functional evidence
rs371587702	p.Thr65Met	1	0.147	Uncertain significance	32.0	Variant with a high predicted potential for a deleterious effect
rs45589337	p.Lys259Glu	9	1.461	Conflicting classifications	21.5	Potential research marker
rs761479700	p.Leu268Val	2	0.299	No definitive clinical classification is available	19.7	Rare variant of uncertain significance

## Data Availability

The datasets generated and analyzed during this study are not publicly available due to ethical restrictions and patient confidentiality protections under Russian Federation laws on personal data protection (Federal Law No. 152-FZ). However, anonymized data supporting the findings may be made available upon reasonable request from qualified researchers, subject to approval by the Local Ethics Committee of the Russian Medical Academy of Continuous Professional Education (contact: rmapo@rmapo.ru). Requests should include a detailed research proposal and data protection plan.

## Supplementary Materials

The following supporting information can be downloaded at: https://www.mdpi.com/article/10.3390/genes17091082/s1, Table S1: Baseline clinical characteristics of the study population.

## Abbreviations

The following abbreviations are used in this manuscript:

Abbreviation	Definition
BAM	Binary Alignment Map
BQSR	Base Quality Score Recalibration
BWA-MEM	Burrows–Wheeler Aligner with Maximal Exact Matches
CADD	Combined Annotation Dependent Depletion
CCDS	Consensus Coding Sequence
CPIC	Clinical Pharmacogenetics Implementation Consortium
DPD	Dihydropyrimidine dehydrogenase
*DPYD*	Dihydropyrimidine dehydrogenase gene
EAS	East Asian
FASTQ	Format for storing nucleotide sequences and their quality scores
FLOT	Fluorouracil, leucovorin, oxaliplatin, and docetaxel
FOLFIRINOX	Folinic acid, fluorouracil, irinotecan, and oxaliplatin
FOLFOX	Folinic acid, fluorouracil, and oxaliplatin
FS	Fisher strand bias
GATK	Genome Analysis Toolkit
GERP	Genomic Evolutionary Rate Profiling
gnomAD	Genome Aggregation Database
GRCh38	Genome Reference Consortium Human Build 38
HGVSc	Human Genome Variation Society coding DNA sequence nomenclature
HGVSp	Human Genome Variation Society protein sequence nomenclature
indel	Insertion or deletion
MANE	Matched Annotation from NCBI and EMBL-EBI
MQ	Mapping quality
mut	Mutant allele
NA	Not available
NFE	Non-Finnish European
PCR	Polymerase chain reaction
QD	Quality by depth
REVEL	Rare Exome Variant Ensemble Learner
SAMtools	Sequence Alignment/Map tools
SIFT	Sorting Intolerant From Tolerant
SNV	Single-nucleotide variant
VCF	Variant Call Format
VEP	Variant Effect Predictor
WES	Whole-exome sequencing
WT	Wild type

## References

[1] White C., Scott R.J., Paul C., Ziolkowski A., Mossman D., Ackland S. (2021). Ethnic Diversity of DPD Activity and the *DPYD* Gene: Review of the Literature. Pharmgenom. Pers. Med..

[2] European Medicines Agency (EMA) EMA Recommendations on DPD Testing Prior to Treatment with Fluorouracil, Capecitabine, Tegafur and Flucytosine. Internet Document: 30 April 2020. https://www.ema.europa.eu/en/news/ema-recommendations-dpd-testing-prior-treatment-fluorouracil-capecitabine-tegafur-flucytosine.

[3] Medicines and Healthcare Products Regulatory Agency 5-Fluorouracil, Capecitabine and Tegafur: DPD Testing Recommended Before Initiation to Identify Patients at Increased Risk of Severe and Fatal Toxicity. Drug Safety Update. Internet Document: 22 October 2020. https://www.gov.uk/drug-safety-update/5-fluorouracil-intravenous-capecitabine-tegafur-dpd-testing-recommended-before-initiation-to-identify-patients-at-increased-risk-of-severe-and-fatal-toxicity.

[4] Lunenburg C.A.T.C., Van Der Wouden C.H., Nijenhuis M., Crommentuijn-van Rhenen M.H., De Boer-Veger N.J., Buunk A.M., Houwink E.J.F., Mulder H., Rongen G.A., Van Schaik R.H.N. (2020). Dutch Pharmacogenetics Working Group (DPWG) Guideline for the Gene–Drug Interaction of *DPYD* and Fluoropyrimidines. Eur. J. Hum. Genet..

[5] García-Alfonso P., Saiz-Rodríguez M., Mondéjar R., Salazar J., Páez D., Borobia A.M., Safont M.J., García-García I., Colomer R., García-González X. (2022). Consensus of Experts from the Spanish Pharmacogenetics and Pharmacogenomics Society and the Spanish Society of Medical Oncology for the Genotyping of *DPYD* in Cancer Patients Who Are Candidates for Treatment with Fluoropyrimidines. Clin. Transl. Oncol..

[6] Deenen M.J., Meulendijks D., Cats A., Sechterberger M.K., Severens J.L., Boot H., Smits P.H., Rosing H., Mandigers C.M.P.W., Soesan M. (2016). Upfront Genotyping of *DPYD***2A* to Individualize Fluoropyrimidine Therapy: A Safety and Cost Analysis. J. Clin. Oncol..

[7] Henricks L.M., Lunenburg C.A.T.C., De Man F.M., Meulendijks D., Frederix G.W.J., Kienhuis E., Creemers G.-J., Baars A., Dezentjé V.O., Imholz A.L.T. (2018). *DPYD* Genotype-Guided Dose Individualisation of Fluoropyrimidine Therapy in Patients with Cancer: A Prospective Safety Analysis. Lancet Oncol..

[8] Amstutz U., Henricks L.M., Offer S.M., Barbarino J., Schellens J.H.M., Swen J.J., Klein T.E., McLeod H.L., Caudle K.E., Diasio R.B. (2018). Clinical Pharmacogenetics Implementation Consortium (CPIC) Guideline for Dihydropyrimidine Dehydrogenase Genotype and Fluoropyrimidine Dosing: 2017 Update. Clin. Pharmacol. Ther..

[9] Meulendijks D., Henricks L.M., Sonke G.S., Deenen M.J., Froehlich T.K., Amstutz U., Largiadèr C.R., Jennings B.A., Marinaki A.M., Sanderson J.D. (2015). Clinical Relevance of *DPYD* Variants c.1679T>G, c.1236G>A/HapB3, and c.1601G>A as Predictors of Severe Fluoropyrimidine-Associated Toxicity: A Systematic Review and Meta-Analysis of Individual Patient Data. Lancet Oncol..

[10] Pratt V.M., Cavallari L.H., Fulmer M.L., Gaedigk A., Hachad H., Ji Y., Kalman L.V., Ly R.C., Moyer A.M., Scott S.A. (2024). *DPYD* Genotyping Recommendations. J. Mol. Diagn..

[11] De Luca O., Salerno G., De Bernardini D., Torre M.S., Simmaco M., Lionetto L., Gentile G., Borro M. (2022). Predicting Dihydropyrimidine Dehydrogenase Deficiency and Related 5-Fluorouracil Toxicity: Opportunities and Challenges of *DPYD* Exon Sequencing and the Role of Phenotyping Assays. Int. J. Mol. Sci..

[12] De Mattia E., Silvestri M., Polesel J., Ecca F., Mezzalira S., Scarabel L., Zhou Y., Roncato R., Lauschke V.M., Calza S. (2022). Rare Genetic Variant Burden in *DPYD* Predicts Severe Fluoropyrimidine-Related Toxicity Risk. Biomed. Pharmacother..

[13] Larrue R., Fellah S., Hennart B., Sabaouni N., Boukrout N., Van Der Hauwaert C., Delage C., Cheok M., Perrais M., Cauffiez C. (2024). Integrating Rare Genetic Variants into *DPYD* Pharmacogenetic Testing May Help Preventing Fluoropyrimidine-Induced Toxicity. Pharmacogenom. J..

[14] Andrews S. (2010). FastQC: A Quality Control Tool for High Throughput Sequence Data. https://www.bioinformatics.babraham.ac.uk/projects/fastqc/.

[15] Chen S., Zhou Y., Chen Y., Gu J. (2018). Fastp: An Ultra-Fast All-in-One FASTQ Preprocessor. Bioinformatics.

[16] Danecek P., Bonfield J.K., Liddle J., Marshall J., Ohan V., Pollard M.O., Whitwham A., Keane T., McCarthy S.A., Davies R.M. (2021). Twelve Years of SAMtools and BCFtools. GigaScience.

[17] McKenna A., Hanna M., Banks E., Sivachenko A., Cibulskis K., Kernytsky A., Garimella K., Altshuler D., Gabriel S., Daly M. (2010). The Genome Analysis Toolkit: A MapReduce Framework for Analyzing next-Generation DNA Sequencing Data. Genome Res..

[18] DePristo M.A., Banks E., Poplin R., Garimella K.V., Maguire J.R., Hartl C., Philippakis A.A., Del Angel G., Rivas M.A., Hanna M. (2011). A Framework for Variation Discovery and Genotyping Using Next-Generation DNA Sequencing Data. Nat. Genet..

[19] Van Der Auwera G.A., O’Connor B.D. (2020). Genomics in the Cloud: Using Docker, GATK, and WDL in Terra.

[20] McLaren W., Gil L., Hunt S.E., Riat H.S., Ritchie G.R.S., Thormann A., Flicek P., Cunningham F. (2016). The Ensembl Variant Effect Predictor. Genome Biol..

[21] Liu X., Li C., Mou C., Dong Y., Tu Y. (2020). dbNSFP v4: A Comprehensive Database of Transcript-Specific Functional Predictions and Annotations for Human Nonsynonymous and Splice-Site SNVs. Genome Med..

[22] Rentzsch P., Witten D., Cooper G.M., Shendure J., Kircher M. (2019). CADD: Predicting the Deleteriousness of Variants throughout the Human Genome. Nucleic Acids Res..

[23] Ioannidis N.M., Rothstein J.H., Pejaver V., Middha S., McDonnell S.K., Baheti S., Musolf A., Li Q., Holzinger E., Karyadi D. (2016). REVEL: An Ensemble Method for Predicting the Pathogenicity of Rare Missense Variants. Am. J. Hum. Genet..

[24] Karczewski K.J., Francioli L.C., Tiao G., Cummings B.B., Alföldi J., Wang Q., Collins R.L., Laricchia K.M., Ganna A., Birnbaum D.P. (2020). The Mutational Constraint Spectrum Quantified from Variation in 141,456 Humans. Nature.

[25] Chan T.H., Zhang J.E., Pirmohamed M. (2024). *DPYD* Genetic Polymorphisms in Non-European Patients with Severe Fluoropyrimidine-Related Toxicity: A Systematic Review. Br. J. Cancer.

[26] Le Teuff G., Cozic N., Boyer J.-C., Boige V., Diasio R.B., Taieb J., Meulendijks D., Palles C., Schwab M., Deenen M. (2024). Dihydropyrimidine Dehydrogenase Gene Variants for Predicting Grade 4-5 Fluoropyrimidine-Induced Toxicity: FUSAFE Individual Patient Data Meta-Analysis. Br. J. Cancer.

[27] Hamzic S., Schärer D., Offer S.M., Meulendijks D., Nakas C., Diasio R.B., Fontana S., Wehrli M., Schürch S., Amstutz U. (2021). Haplotype Structure Defines Effects of Common *DPYD* Variants c.85T > C (Rs1801265) and c.496A > G (Rs2297595) on Dihydropyrimidine Dehydrogenase Activity: Implication for 5-fluorouracil Toxicity. Br. J. Clin. Pharmacol..

[28] Deenen M.J., Tol J., Burylo A.M., Doodeman V.D., De Boer A., Vincent A., Guchelaar H.-J., Smits P.H.M., Beijnen J.H., Punt C.J.A. (2011). Relationship between Single Nucleotide Polymorphisms and Haplotypes in *DPYD* and Toxicity and Efficacy of Capecitabine in Advanced Colorectal Cancer. Clin. Cancer Res..

[29] Fernandez-Rozadilla C., Cazier J.B., Moreno V., Crous-Bou M., Guinó E., Durán G., Lamas M.J., López R., Candamio S., Gallardo E. (2013). Pharmacogenomics in Colorectal Cancer: A Genome-Wide Association Study to Predict Toxicity after 5-Fluorouracil or FOLFOX Administration. Pharmacogenom. J..

[30] Boige V., Vincent M., Alexandre P., Tejpar S., Landolfi S., Le Malicot K., Greil R., Cuyle P.J., Yilmaz M., Faroux R. (2016). *DPYD* Genotyping to Predict Adverse Events Following Treatment with Fluorouracil-Based Adjuvant Chemotherapy in Patients with Stage III Colon Cancer: A Secondary Analysis of the PETACC-8 Randomized Clinical Trial. JAMA Oncol..

[31] Ruzzo A., Graziano F., Galli F., Galli F., Rulli E., Lonardi S., Ronzoni M., Massidda B., Zagonel V., Pella N. (2017). Dihydropyrimidine Dehydrogenase Pharmacogenetics for Predicting Fluoropyrimidine-Related Toxicity in the Randomised, Phase III Adjuvant TOSCA Trial in High-Risk Colon Cancer Patients. Br. J. Cancer.

