# Supplementary material for: Allelic Variants of the DPYD Gene in Russian Patients with Cancer: The Results of Exome Sequencing
Source: Genes (Basel). 2026 Sep 9;17(9):1082. doi: 10.3390/genes17091082 (PMC13606897; doi:10.3390/genes17091082)
Supplement: Supplementary file 1 [file genes-17-01082-s001.zip › genes-4554769-supplementary.pdf]

Supplementary Table S1. Baseline clinical characteristics of the study population

Characteristic	Value
Total number of patients	339
Age, years	
Mean ± SD	64.5 ± 10.3
Median (IQR)	66 (59-72)
Range	32-84
Sex, n (%)	
Male	192 (56.6%)
Female	147 (43.4%)
Cancer type, n (%)	
Colon cancer	129 (38.1%)
Gastric cancer	123 (36.3%)
Rectal cancer	45 (13.3%)
Pancreatic cancer	39 (11.5%)
Small bowel cancer	2 (0.6%)
Esophageal cancer	1 (0.3%)
Tumor sidedness among colorectal cancer patients, n = 174	
Right-sided	39 (22.4%)
Left-sided	135 (77.6%)
Disease stage, n (%)	
Stage I	11 (3.2%)
Stage II	44 (13.0%)
Stage III	120 (35.4%)
Stage IV	164 (48.4%)
Histological differentiation, n (%)	
G1-G2	198 (58.4%)
G3-G4	100 (29.5%)
Not assessed	41 (12.1%)
ECOG performance status, n (%)	
ECOG 0	96 (28.3%)
ECOG 1	217 (64.0%)
ECOG 2	26 (7.7%)
Comorbidities, n (%)	
Any concomitant disease	268 (79.1%)
Arterial hypertension	201 (59.3%)
Diabetes mellitus	61 (18.0%)
Ischemic heart disease	55 (16.2%)
Arrhythmia	29 (8.6%)
History of stroke	21 (6.2%)
Thyroid disease	19 (5.6%)
Polymorbidity	62 (18.3%)
Lifestyle and family history, n (%)	
Smoking, yes	94 (27.7%)
Family history of cancer	125 (36.9%)
Family history of gastrointestinal cancer	97 (28.6%)
Nutritional characteristics	
Weight loss during the preceding ≥3 months, kg, median (IQR)	9.0 (5.0-14.0)
Weight loss during the preceding ≥3 months, % of baseline weight, median (IQR)*	10.53 (5.75-15.78)
Treatment setting, n (%)	
Neoadjuvant chemotherapy	76 (22.4%)

First-line chemotherapy	181 (53.4%)
Adjuvant chemotherapy	82 (24.2%)
